# Comparison of Pericapsular Nerve Group (PENG) Block Versus Quadratus Lumborum (QL) Block for Analgesia After Primary Total Hip Arthroplasty Under Spinal Anesthesia: A Retrospective Study

**DOI:** 10.7759/cureus.50119

**Published:** 2023-12-07

**Authors:** Andrew S Braun, Jacelyn E Peabody Lever, Hari Kalagara, Paul D Piennette, Sivasenthil Arumugam, Scott Mabry, Kesha Thurston, Sameer Naranje, Joel Feinstein, Promil Kukreja

**Affiliations:** 1 Anesthesiology and Perioperative Medicine, University of Alabama at Birmingham (UAB), Birmingham, USA; 2 Anesthesiology and Perioperative Medicine, Heersink School of Medicine, University of Alabama at Birmingham (UAB), Birmingham, USA; 3 Department of Anesthesiology and Perioperative Medicine, Mayo Clinic, Jacksonville, USA; 4 Anesthesiology, University of Connecticut School of Medicine, Farmington, USA; 5 Anesthesiology, Saint Francis Hospital and Medical Center / University of Connecticut, Hartford, USA; 6 Orthopaedics, University of Alabama at Birmingham (UAB), Birmingham, USA; 7 Anesthesiology, University of Alabama at Birmingham (UAB), Birmingham, USA

**Keywords:** postoperative analgesia, regional nerve blocks, acute pain management, oral morphine equivalents, total hip arthroplasty (tha), anesthesia spinal, quadratus lumborum block, pericapsular nerve group (peng) block

## Abstract

Background: Total hip arthroplasty (THA) is one of the most common operative procedures performed. Controlling postoperative pain following THA remains a challenge due to the complex innervation of the hip joint and the recent desire to preserve motor function following nerve blockade. Several nerve block techniques have been used for THA in the past, but the quadratus lumborum (QL) block and the blockade of the pericapsular nerve group (PENG) have emerged as opiate-sparing regional anesthesia techniques that preserve motor function. To date, little data comparing the two block techniques exists. The purpose of our study was to compare outcomes following these techniques in patients undergoing primary THA.

Materials and methods: This retrospective analysis utilized data from three distinct groups who underwent primary THA at our institution: 45 patients who received PENG block, 38 patients who received QL block, and 77 control patients. Chart review analysis was performed by authorized personnel to obtain cumulative oral morphine equivalent (OME) data at 24 and 48 hours postoperatively (primary outcomes). In addition, visual analog pain scale (VAS) scores in the post-anesthesia care unit (PACU) and at 12, 24, and 48 hours, ambulation distance, and length of hospital stay data were obtained (secondary outcomes). Group comparisons were conducted using either analysis of variance (ANOVA) with Tukey’s multiple comparison test for parametric data or Krustal-Wallis with Dunn’s multiple comparison tests for nonparametric endpoints.

Results: This study found a statistically significant difference in cumulative OME usage across all groups at 24 and 48 hours. Significant difference in OMEs was found between QL and control and PENG and control; however, no difference was found in OMEs between PENG and QL groups at either time point. There was a statistically significant difference in VAS scores in the PACU across all groups; QL showed significantly lower VAS scores in the PACU compared to PENG and control, while PENG only showed significantly lower VAS scores compared to control. There was a statistically significant difference in VAS scores at 24 hours across all groups; however, only QL showed significantly lower VAS scores compared to control at 24 hours. QL was associated with a statistically significant increase in the length of hospital stay compared to PENG.

Conclusion: This study showed no difference between OME usage in patients who received PENG or QL nerve blocks for primary THA. VAS scores were similar between groups with the exception of QL outperforming PENG in the PACU. Optimizing postoperative pain via multi-approach strategies should remain a priority for patients undergoing THA. Future research is warranted in order to provide guidance on best practice for these patients.

## Introduction

As the elderly and obese populations continue to grow in the United States, osteoarthritis (OA) remains a prevalent cause of debilitation and diminished quality of life. Total hip arthroplasty (THA) has become increasingly popular as a means to alleviate pain for patients living with hip joint arthritis. Due to its ability to significantly improve quality of life, THA has emerged as one of the most frequently performed surgical procedures [1-3]. Since the introduction of prosthetic hips in the 1930s, advancements in technology and surgical technique have afforded patients earlier mobilization, improved stability, and faster time to discharge postoperatively [3]. Furthermore, recent emphasis on enhanced recovery pathways post-surgery has led to the integration of opiate-sparing multimodal pain strategies, regional anesthesia techniques, and early postoperative physical therapy.

Several regional anesthesia techniques have been described as safe and efficacious for THA. However, methods, such as lumbar plexus block and femoral nerve block, have fallen out of favor for THA due to the more recent desire to preserve postoperative motor strength. Novel approaches, such as the quadratus lumborum (QL) Block and blockade of the pericapsular nerve group (PENG), have emerged as regional approaches that ensure the proper balance of adequate analgesia and intact motor strength following THA.

The transmuscular QL block is a relatively new technique in which a local anesthetic is deposited in the fascial plane between the QL and the psoas major muscles, with the expectation that it will spread to the thoracic paravertebral space and to the branches of the lumbar plexus [4,5]. A recent cadaveric study and case series concluded that the supra-iliac approach to the anterior QL block provided T10-L3 dermatomal coverage and provided effective analgesia for hip surgery [6]. Recent publications support the efficacy of QL for THA, noting its association with reduced morphine equivalents, enhanced functional recovery, improved mobility following surgery, and reduced hospital length of stay [4,7-9].

The PENG block targets articular branches of the femoral, obturator, and accessory obturator nerves, providing analgesia for patients undergoing THA [10,11]. A local anesthetic is injected into the anterior hip capsule, which lies in the fascial plane between the psoas tendon and iliopubic eminence in the pelvis. The femoral and obturator nerves from the lumbar plexus innervate the anterolateral and anteromedial hip capsules, respectively [12,13]. This approach has been shown to reduce opiate usage, lower pain scores, and improve quality of recovery for THA patients in randomized control trials (RCTs) [14-16]. Both the QL and PENG blocks are credited with enhancing timely participation in physical therapy following surgery compared to traditional nerve blocks.

Despite the positive outcomes associated with these regional block techniques, the equipoise between the QL and PENG techniques leaves physicians uncertain regarding the best practice. Retrospective data suggest combining these approaches for optimal analgesia [17], but direct comparisons in the literature for primary THA are limited. Recent publications have provided insights on this comparison, with Wang et al. finding lower pain scores in the PENG group immediately postoperatively, but no difference in opiate consumption compared to QL in an RCT [18]. Another RCT comparing PENG, QL, and local infiltration analgesia (LIA) techniques for patients undergoing THA with spinal anesthesia found similar analgesic benefit between PENG and QL [19].

The purpose of our study was to directly examine the outcomes of patients undergoing THA with spinal anesthesia who received preoperative QL or PENG blocks. Due to the lack of a gold standard for the best regional approach to THA, the choice of block at our institution is variable. We hypothesized that the QL block would outperform the PENG block with regard to pain outcomes due to its broad dermatomal coverage and known analgesic benefit over control. In addition, we hypothesized that both nerve blocks would offer benefit over patients who received no block.

## Materials and methods

Study approval and patient selection

This study was granted approval by our Institutional Review Board (University of Alabama at Birmingham, IRB #300000976). This retrospective, single-institution study utilized data from patients undergoing primary THA who received a QL or PENG block compared to no block. In total, we examined data from 45 patients who received the PENG block, 38 patients who received the QL block, and 77 patients who received no regional block. All regional blocks were performed in our institution’s preoperative area following the administration of 1000 milligrams of acetaminophen and 200-400 milligrams celecoxib. All PENG blocks were performed with the patient in the supine position, and all QL blocks were performed in the lateral decubitus position. All patients in these studies received spinal anesthesia with 12-15 milligrams bupivacaine. All patients reviewed in this study were age 18 and older and had American Society of Anesthesiologists (ASA) physical status class 1-3. Patients who were excluded were ASA class 4 or higher, had allergies or any intolerance to local anesthesia, had pre-existing neurologic or anatomic deficits, had preoperative opiate tolerance (chronic, daily use of 60 oral morphine equivalents or greater), or had coexisting coagulopathy or anticoagulant use.

Procedure details

Ultrasound-Guided Technique for the QL Block

**Figure 1:**
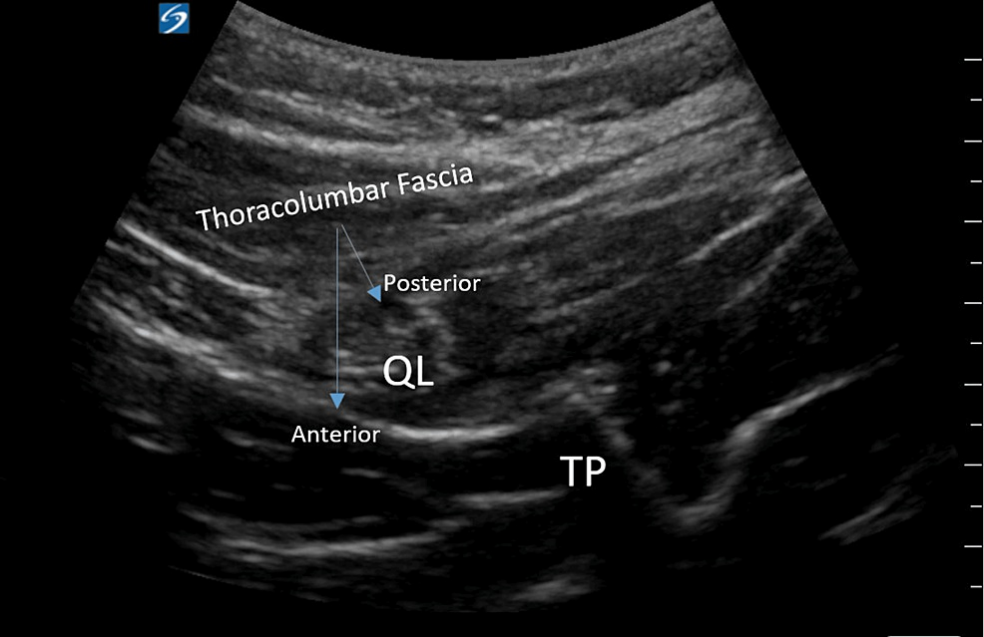
Ultrasound-guided technique for the quadratus lumborum block QL, quadratus lumborum; TP, transverse process

A low-frequency, curvilinear transducer was placed midline in order to identify spinous processes at the levels of L3 and L4. The probe was then translocated in a lateral fashion until the transverse process (TP) of L4 was visualized with the QL muscle between the erector spinae muscle group (posterior) and the psoas major (anterior). A hyperechoic needle was then inserted in-plane from posterior to anterior, and a local anesthetic was deposited in the fascial plane between the QL and psoas major muscles (Figure 1).

Ultrasound-Guided Technique for the PENG Block

**Figure 2:**
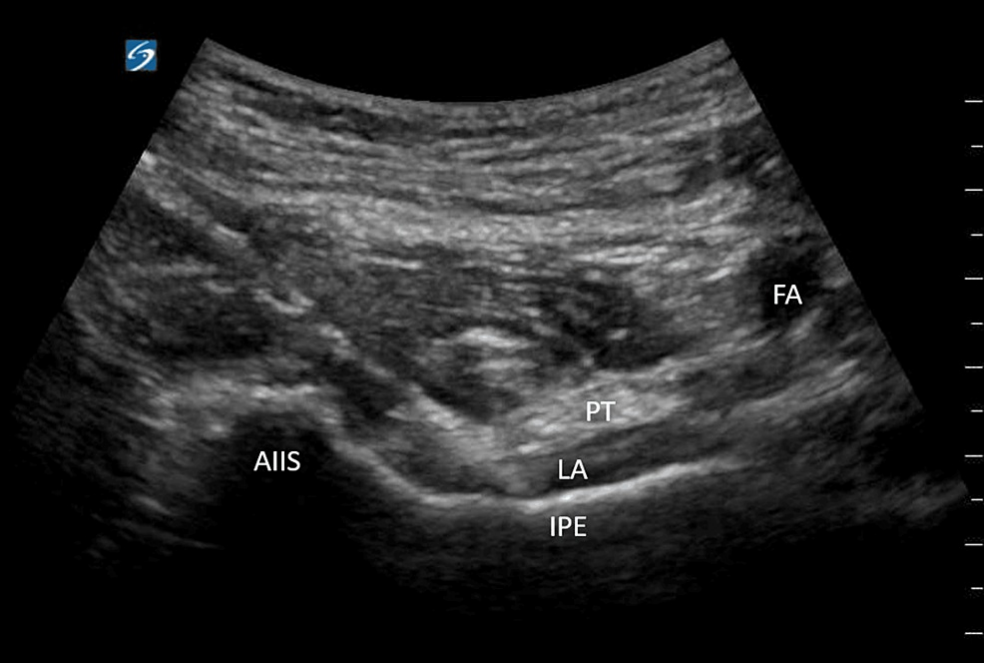
Ultrasound-guided technique for the PENG block AIIS, anterior inferior iliac spine; PT, psoas tendon; FA, femoral artery; IPA, iliopubic eminence; LA, local anesthetic; PENG: pericapsular nerve group

A low-frequency, curvilinear transducer was placed in a transverse fashion over the anterior inferior iliac spine (AIIS) and translocated inferiorly and medially to visualize the iliopubic eminence, psoas tendon, and femoral artery. A hyperechoic needle was advanced in-plane from lateral to medial, and a local anesthetic was deposited between the psoas tendon and the iliopubic eminence (Figure 2).

Data Collection, Statistical Analysis, and Data Presentation

The primary outcomes for this study included oral morphine equivalents (OMEs) in 24 and 48 hours. Secondary outcomes included visual analog pain scale (VAS) scores in the post-anesthesia care unit (PACU) and at 12, 24, and 48 hours, length of hospital stay, and ambulation distance at 24 hours. An authorized personnel conducted chart review in the medical record to extract outcome data for this retrospective study per IRB-approved protocol. Data are presented as mean and standard error of the mean for continuous variables or number and percentage of total for categorical variables. Group comparisons were conducted using either ANOVA with Tukey’s multiple comparison test for parametric data or Krustal-Wallis with Dunn’s multiple comparison tests for nonparametric endpoints. A p-value of less than 0.05 was considered statistically significant. All statistical analyses and graph generation were carried out using Graphpad Prism version 9.3.1 for Mac OS X (Graphpad, Boston, MA).

Manuscript Preparation and Figure Generation

The manuscript was originally drafted in Microsoft Word Processor (Microsoft, Redmond, WA). Large language model tools (OpenAI, San Francisco, CA; Grammarly, San Francisco, CA) were used for assistance in the editing stage of the manuscript preparation.

## Results

Our retrospective study compared three distinct patient groups who underwent primary THA and received either a PENG or QL block for postoperative analgesia or received no block. Both groups were compared to a control group (no block) and to each other. Primary outcome measures for this study were the cumulative amount of OMEs used in 24 and 48 hours following surgery. Secondary outcomes included length of time from nerve block to discharge, VAS scores during admission, and distance ambulated at 24 and 48 hours. The data were extracted through a chart review (see Table 1 for patient demographics). Outcome measures for the three groups for this study (control, PENG, and QL) are summarized in Table 2.

**Table 1:** Demographics of the study patients. For race/ethnicity and sex, the displayed values are as follows: number of study patients (percentage of study patients). For age, body mass index, and American Society of Anesthesiologists (ASA) score, the displayed values are as follows: mean value (standard error of the mean (SE)). QL, quadratus lumborum; PENG, pericapsular nerve group

Demographic	Control (n=77)	PENG (n = 45)	QL (n = 38)
Age, mean (SE)	61.9, (1.2)	56.1, (2.2)	58.7, (2.7)
Race/ethnicity, N (%)			
African American	35 (45%)	17 (38%)	19 (50%)
White	42 (54%)	28 (62%)	19 (50%)
Sex, N (%)			
Female	39 (51%)	25 (55%)	21 (55%)
Male	38 (49%)	20 (45%	17 (45%)
Body mass index, mean (SE)	30.9 (0.6)	30.8 (0.96)	29.6 (0.98)
ASA score, mean (SE)	2.8 (0.04)	2.6 (0.08)	2.6 (0.08)

**Table 2:** Summary of the primary and secondary outcomes. The displayed values are as follows: mean value (standard error of the mean (SE)). A p-value of less than 0.05 was considered statistically significant, as indicated by *. QL, quadratus lumborum; PENG, pericapsular nerve group; OME, oral morphine equivalent; PACU, post-anesthesia care unit

Outcome	Control (n=77)	PENG (n = 45)	QL (n = 38)	P*
OME, mean (SE)				
24 hours (cumulative)	52.7 (4.3)	38.3 (4.4)	27.7 (3.5)	<0.001
48 hours (cumulative)	72.9 (6.1)	50.6 (6.3)	44.6 (6.1)	0.004
Pain score, mean (SE)				
PACU	1.5 (0.2)	1.8 (0.2)	0.1 (0.1)	<0.0001
12 hours	2.4 (0.3)	1.9 (0.4)	2.2 (0.4)	0.707
24 hours	3.2 (0.3)	3.1 (0.4)	2.2 (0.4)	0.036
48 hours	3.2 (0.4)	2.2 (0.5)	3.8 (0.5)	0.128
Ambulation distance (feet) mean (SE)				
24 hours	98.0 (7.7)	118.1 (11.1)	113.4 (10.5)	0.229
48 hours	89.9 (12.4)	78.6 (14.7)	128.8 (18.3)	0.142
Length of stay (hours), mean (SE)	41.7 (2.4)	34.2 (2.8)	52.5 (4.8)	0.002

The data for this study were extracted from two previously conducted and completed prospective, randomized studies [4,14]. To evaluate the analgesic efficacy of PENG and QL blocks, we performed an analysis of the electronic Medication Administration Record (MAR) to examine the distribution of pro re nata (PRN) opioid medication over time (Figure 1). The cumulative OMEs were calculated for patients during their stay at 24 and 48 hours postoperatively.

**Figure 3:**
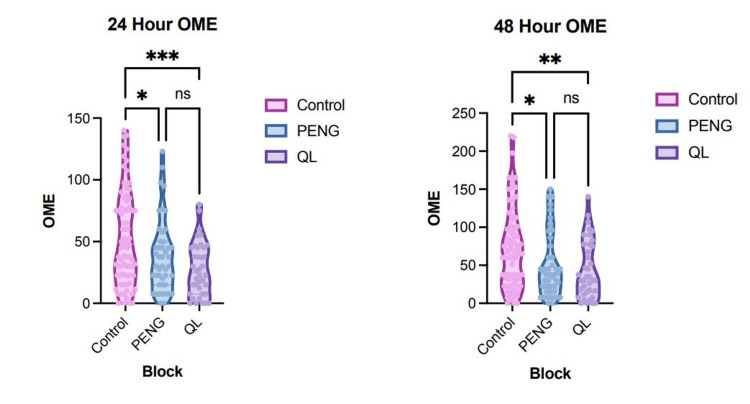
Opioid consumption. A (left): 24-hour cumulative OME use. B (right): 48-hour cumulative OME use. A p-value ≤ 0.05 was considered statistically significant. Values that are not statistically significant are indicated by "ns." * indicates p-value ≤ 0.05; ** indicates p-value ≤ 0.01; *** indicates p-value ≤ 0.001; **** indicates p-value ≤ 0.0001. QL, quadratus lumborum; PENG, pericapsular nerve group; OME, oral morphine equivalent

The computation of cumulative OMEs within the initial 24 hours postoperatively revealed significant differences. Cumulative OME use at 24 hours was significantly different across the three groups (p=0.0004). Both the PENG and QL groups had substantially lower cumulative OMEs at 24 hours compared to control; the mean difference for PENG vs. control was 14.4 OME (95% confidence interval (CI) of difference 0.03-28.8, p=0.0495), and the mean difference for QL vs. control was 24.98 OME (95% CI of difference 9.78-40.17, p=0.0004). Notably, OME use was not different between the QL and PENG groups at 24 hours, with a mean difference of 10.6 OME (95% CI of difference -27.5-6.31, p=0.303) (Figure 3A).

Similar findings were observed at 48 hours. Cumulative OME use at 48 hours was significantly different (p=0.004). Both the PENG and QL groups had substantially lower cumulative OMEs at 48 hours compared to control; the mean difference for PENG vs. control was 22.2 OME (95% CI of difference 1.23-43.24, p=0.035), and the mean difference for QL vs. control was 28.23 OME (95% CI of difference 6.04-50.43, p=0.0085). Notably, OME use was not different at 48 hours between the PENG and QL groups, with a mean difference of 6.0 OME (95% CI of difference -18.67-30.66, p=0.834) (Figure 3B).

**Figure 4:**
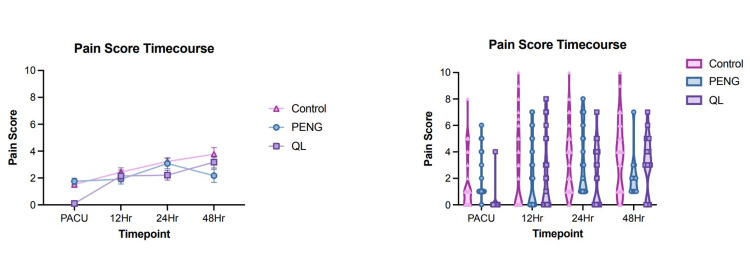
VAS pain scores. A (left): Pain score time course longitudinally through 48 hours following surgery. B (right): Pain score time course by group at the examined time points. VAS, visual analog pain scale; QL, quadratus lumborum; PENG, pericapsular nerve group; PACU, post-anesthesia care unit

VAS scores were tracked longitudinally up to 48 hours postoperatively following THA (Figure 4A). Statistical analysis of the PACU VAS scores indicated a significant difference (p<0.0001), with QL demonstrating the lowest scores. The mean VAS scores (95% CI) in the PACU were 1.52 (1.08-1.95) for control, 1.76 (1.29-2.22) for PENG, and 0.11 (-0.11-0.32) for QL. The QL block group had significantly lower VAS scores compared to both control and PENG, both with p<0.0001. PENG also had a significantly lower VAS scores compared to control (p=0.036). No significant differences were noted between groups at 12 hours (p=0.708); the mean VAS scores (95% CI) at 12 hours were 2.44 (1.79-3.08) for control, 1.91 (1.18-2.64) for PENG, and 2.16 (1.27-3.04) for QL. The mean VAS scores (95% CI) at 24 hours were 3.22 (2.68-3.77) for control, 3.08 (2.29-3.87) for PENG, and 2.21 (1.47-2.95) for QL. VAS scores were significantly lower for only QL compared to control at 24 hours (p=0.036). Control vs. PENG was not statistically significantly different (p>0.999). No significant differences were noted between the groups at 48 hours (p=0.128); the mean VAS scores (95% CI) at 48 hours were 3.18 (2.27-4.10) for control, 2.17 (1.09-3.25) for PENG, and 3.77 (2.79-4.76) for QL. Figure 4B displays the pain score time course by group.

**Figure 5:**
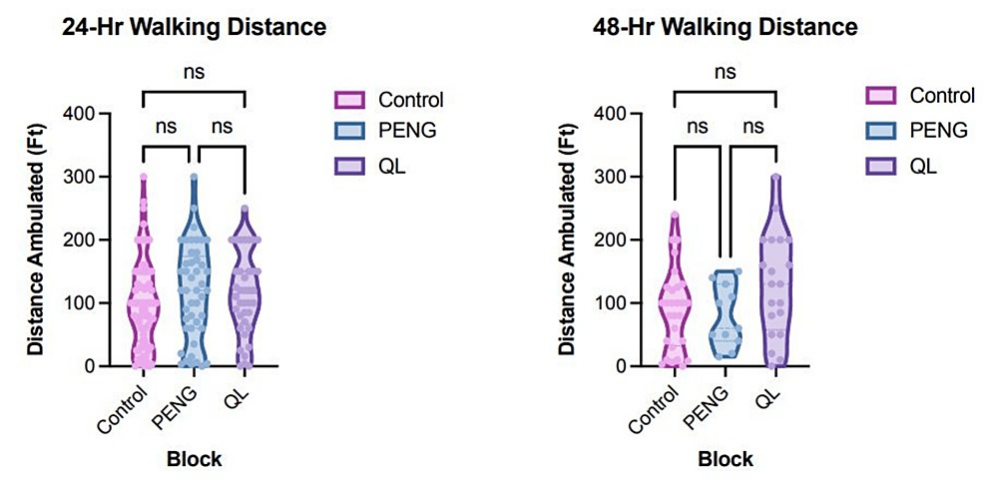
Ambulation distance walked. A (left): Distance walked 24 hours following surgery. 5B (right): Distance walked 48 hours following surgery. A p-value ≤0.05 was considered statistically significant. Values that are not statistically significant are indicated by "ns." QL, quadratus lumborum; PENG, pericapsular nerve group

Figure 5 focuses on the ambulation distance in feet at two key time points: 3A) 24 hours and 3B) 48 hours postoperatively. At the 24-hour mark, there was no statistically significant differences in the ambulation distance among the three groups (p=0.219). The mean (SEM) distances were as follows: control 98.03 feet (7.72), PENG 118.1 feet (11.14), and QL 113.4 feet (10.5). Dunn's post-hoc tests further confirmed the absence of significant differences between PENG and QL (p>0.999), PENG and control (p=0.336), and QL and control (p=0.664). Similarly, at the 48-hour mark, despite a trend indicated by the Kruskal-Wallis test (p=0.142), there were no statistically significant differences in ambulation distance. The mean (SEM) distances in feet were as follows: control 89.89 feet (12.39), PENG 78.64 feet (14.74), and QL 128.8 feet (18.32). Dunn's post-hoc tests again did not reveal significant differences between PENG and QL (p=0.361), PENG and control (p>0.999), and QL and control (p=0.220).

**Figure 6:**
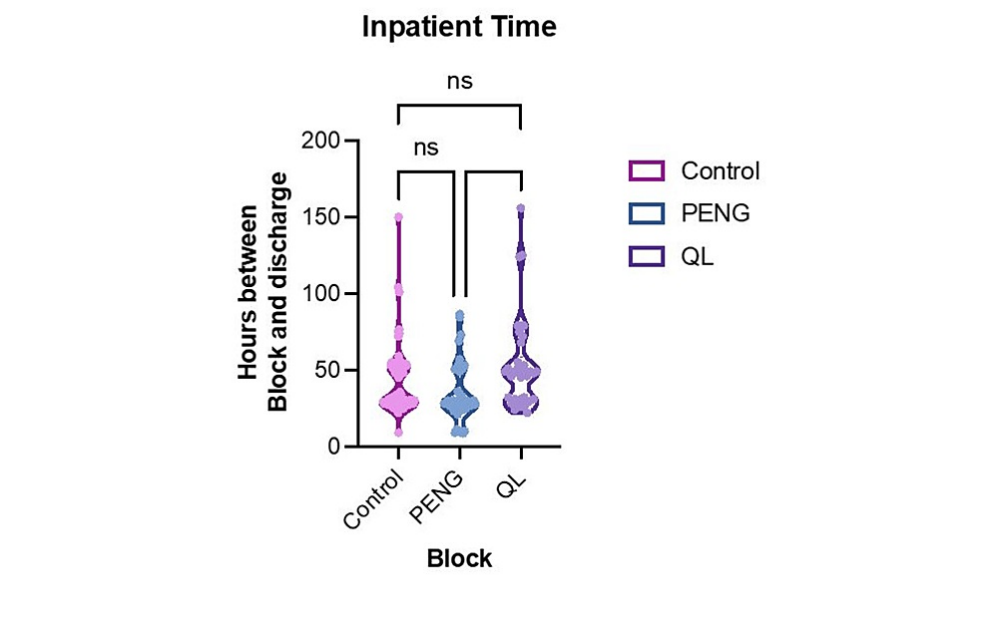
Length of stay following the regional nerve block procedure. A p-value ≤0.05 was considered statistically significant. Values that are not statistically significant are indicated by "ns." QL, quadratus lumborum; PENG: pericapsular nerve group

Figure 6 provides information on the duration from nerve block to discharge. This indicated a significant difference among the groups (p=0.002). The mean (SEM) inpatient times were as follows: control 41.7 hours (2.4), PENG 34.2 hours (2.8), and QL 52.5 hours (4.8). Dunn's post-hoc tests unveiled a significant difference between PENG and QL (p=0.0015), a trend between PENG and control (p=0.064), and no significant difference between QL and control (p=0.26).

## Discussion

THA is a life-altering orthopedic surgery seeing an increased demand. The ideal peripheral nerve block for THA has proved challenging to determine based on the complex innervation of the hip and the desire to avoid postoperative weakness. The goal of our study was to find meaningful differences in two of the modern, motor-sparing regional anesthesia techniques in order to provide guidance for best practices for these patients. We found that the patients who received QL blocks for THA had significantly better pain control in the PACU compared to those who received PENG blocks; however, no significant difference was found in subsequent pain scores or OME usage at any time point. Both groups had significantly better pain outcomes compared to patients who received no nerve blocks.

The PENG block is a relatively novel regional technique for hip analgesia. There are few recent RCTs studying this block for THA [15-16,20-21]. Three studies assessed the benefit of adding a PENG block when patients in both groups had LIA. Of these, only a study by Pascarella et al. supports using the PENG blocks for THA. Pascarella found that pain scores were significantly lower in patients receiving PENG blocks at all time points. The median (interquartile range (IQR)) was 3 (2.0-4.0) for the PENG group and 6 (5.0-6.0) in the control group at 24 hours. Furthermore, the PENG group had reduced opioid consumption, improved hip range of motion, and shorter time to ambulation [15].

Although our retrospective study did not directly measure motor weakness, a prior RCT demonstrated less motor weakness with the PENG block when compared to the suprainguinal fascia iliaca nerve block technique [20]. This study also found no difference in postoperative pain scores or cumulative OMEs comparing the PENG block to the suprainguinal fascia iliaca block [20]. In another study, patients who received PENG blocks for THA had improved pain scores compared to femoral nerve blocks [21]. While our results did not achieve statistical significance, some observed trends are noteworthy: the PENG group was associated with a decreased length of stay and improved ambulation at the 24-hour time point. These trends merit further investigation due to their potential clinical and economic significance. It is also worth noting that the PENG block may offer worthwhile improvement in patients' block experience as it can be performed in the supine position. This may be relevant when considering patients with contralateral hip or shoulder pain.

Although the efficacy of LIA for total knee arthroplasty (TKA) surgery is well established, the efficacy of LIA for THA is less certain [22]. The 2019 American Association of Hip and Knee Surgeons (AAHKS) survey showed that approximately 80% of respondents used periarticular injection or local infiltration anesthesia for both TKA and THA patients [23]. Our institution does not routinely use LIA; thus, comparing PENG and QL without this intervention helps to establish the effect size of the nerve block itself.

The main regional techniques for THA include lumbar plexus block, lumbar epidural, femoral nerve block, sciatic nerve block, fascia iliaca block, pericapsular injection, or obturator nerve block. Unfortunately, these blocks have been known to provide inconsistent or partial analgesia, and some are associated with lower extremity weakness that may interfere with mobility and balance. One study examining continuous femoral nerve blocks for THA found opioid requirements of approximately 160mg of OMEs; an alternate study of continuous lumbar plexus block showed approximately 114 mg of OMEs in the first 48 hours [24]. A prospective, randomized study showed that the 48-hour OME consumption in the QL block group was 54±8 mg, which is significantly lower than the two aforementioned regional block techniques [4]. This study also found superior analgesia following QL blocks for primary THA, resulting in lower pain scores and less opioid consumption with no difference in distance walked during PT when compared with control [4]. 

In our study, patients who received QL or PENG blocks experienced better pain control and showed reduced opioid consumption when compared to patients who received no block. It is important to note that those in the QL block group showed trends of less OME usage and lower VAS scores compared to the PENG group. This can be explained by the broader dermatonmal coverage achieved with QL blocks compared to PENG [8]. Our study also found no statistically significant difference in ambulation distances at 24 and 48 hours between block and control, which could imply that the block patients had equal ability to participate in physical therapy as those who received no block.

There are some limitations to this retrospective study. First, we did not assess quadriceps muscle strength objectively; thus, we could not address the potential spread of local anesthetics to the lumbar plexus after the anterior QL block approach. Second, data were not stratified for the surgical approach, which may affect the severity of postoperative pain and functional recovery. Third, we did not perform any sensory or motor testing as the PENG block is not known to affect cutaneous nerves, and motor testing is challenging in the immediate postoperative setting. Fourth, we included only ASA 1-3 and opioid-naïve patients having spinal anesthesia to reduce the effect of the anesthetic technique on the quality of recovery. Therefore, our results may not be generalizable to opioid-tolerant patients or patients receiving general anesthesia. Finally, this study examined patients from a single institution. Larger-scale studies that include patients from multiple institutions would again improve the reproducibility of our results.

## Conclusions

The complex innervation of the hip and the desire to preserve motor function postoperatively creates a unique challenge when attempting to define a single, best regional anesthesia technique for THA. Our retrospective study found lower OME usage in patients who received either PENG or QL blocks compared to no block. We also found improved PACU pain scores in patients who received QL blocks compared to PENG, but no difference in OME usage was found between the groups. Future RCTs and meta-analyses are needed in order to clarify the opiate sparing role of regional anesthesia in THA and find consensus on best practices.
